# Oxytocin Facilitates Social Learning by Promoting Conformity to Trusted Individuals

**DOI:** 10.3389/fnins.2019.00056

**Published:** 2019-02-06

**Authors:** Lei Xu, Benjamin Becker, Keith M. Kendrick

**Affiliations:** The Clinical Hospital of Chengdu Brain Science Institute, MOE Key Laboratory for NeuroInformation, University of Electronic Science and Technology of China, Chengdu, China

**Keywords:** oxytocin – therapeutic use, social conformity, interpersonal trust, expert, social learning

## Abstract

There is considerable interest in the role of the neuropeptide oxytocin in promoting social cohesion both in terms of promoting specific social bonds and also more generally for increasing our willingness to trust others and/or to conform to their opinions. These latter findings may also be important in the context of a modulatory role for oxytocin in improving the efficacy of behavioral therapy in psychiatric disorders. However, the original landmark studies claiming an important role for oxytocin in enhancing trust in others, primarily using economic game strategies, have been questioned by subsequent meta-analytic approaches or failure to reproduce findings in different contexts. On the other hand, a growing number of studies have consistently reported that oxytocin promotes conformity to the views of groups of in-group individuals. Most recently we have found that oxytocin can increase acceptance of social advice given by individual experts without influencing their perceived trustworthiness *per se*, but that increased conformity in this context is associated with how much an expert is initially trusted and liked. Oxytocin can also enhance the impact of information given by experts by facilitating expectancy and placebo effects. Here we therefore propose that a key role for oxytocin is not in facilitating social trust *per se* but in conforming to, and learning from, trusted individuals who are either in-group members and/or perceived experts. The implications of this for social learning and use of oxytocin as an adjunct to behavioral therapy in psychiatric disorders are discussed.

## Introduction

Interpersonal trust within social groups is of key importance for social interactions, bonds, cooperation and learning and trust between different groups can also help ensure a stable and peaceful co-existence as well as mutually beneficial co-operation and trade. Trust is generally considered to be critical for co-operation and reciprocity in social and economic interactions but importantly trust also involves risk of potential injury if misplaced or broken and we have a natural aversion to taking such risks (Hardin, 2002; Ostrom and Walker, 2003). Indeed, an important factor influencing our trust behavior is that we are strongly motivated to avoid others betraying our trust (Bohnet and Zeckhauser, 2004; Bohnet et al., 2008). Trust can potentially be influenced by our assessment of the level of risk that trusting others might have and also by increased sensitivity to physical and/or other cues for detecting trustworthiness. It is therefore of great importance to identify both behavioral and physiological factors which can act to enhance trust, particularly in situations where individuals have impaired trust and therefore find it hard to interact socially with others and learn from them and/or to benefit optimally from cognitive and behavioral therapeutic strategies.

## Oxytocin and Interpersonal Trust in the Context of Economic Games

For more than a decade the potential role of the hypothalamic neuropeptide oxytocin (OXT) in enhancing interpersonal trust in humans has received considerable attention. In an initial landmark study, Kosfeld et al. (2005) first reported that intranasal OXT administration could increase trust toward others in terms of being willing to make higher risk investments. This suggested that OXT might make individuals less risk-averse to trusting others. Following on from this Baumgartner et al. (2008) reported that intranasal OXT influenced the neural circuitry involved in trust and adaptation in response to it, notably in the amygdala and striatum critically engaged in fear and adaptive learning as well as the modulation of these functions by social contexts.

Subsequently it was reported that claimed trust-promoting effects of OXT in the context of economic games were sensitive to previous experience in that they did not occur following trust betrayal (Mikolajczak et al., 2010a), although this was contrary to the findings of Baumgartner et al. (2008) where experience of trust betrayal was found to have no effect on trust behavior after intranasal OXT. Another study reported that following experience of unfair treatment in the trust game women, but not men, were subsequently less forgiving of unfair treatment after intranasal OXT (Yao et al., 2014), which also suggested that it was not promoting “blind-trust.” However, although an initial meta-analysis of OXT effects on trust reported a modest effect size (Van IJzendoorn and Bakermans-Kranenburg, 2012) a more recent review and meta-analysis restricted to studies involving economic games failed to demonstrate overall significant effects of OXT on interpersonal trust *per se*, thereby casting doubt on its role (Nave et al., 2015). Recently, another small-scale within subject study has actually reported some evidence for reduced trust in the context of a multi-round trust game following intranasal OXT (Ide et al., 2018).

Genetic association studies have additionally provided some support for a role of OXT and its receptor (OXTR) in trust during economic game paradigms (Israel et al., 2009; Krueger et al., 2012), although another study (Apicella et al., 2010) and a subsequent meta-analysis (see Bakermans-Kranenburg and van IJzendoorn, 2014) failed to confirm overall significance of these findings. While some studies have reported associations between trust/trustworthiness and blood concentrations of OXT (Zak et al., 2005; Zhong et al., 2012) others have not (Christensen et al., 2014) and some positive findings need to be treated with caution where unextracted assay protocols were employed which may not be reliable (see Leng and Ludwig, 2016).

There has been some other indirect support for a potential role of OXT in influencing interpersonal trust in economic games. Thus, testosterone treatment which would normally interact negatively with OXT (Crespi, 2016) was found to decrease trust in economic games (Bos et al., 2010). On the other hand, enhancing serotonin function using treatment with tryptophan, which would be expected to indirectly enhance OXT signaling (Dölen et al., 2013), increased trust in the same context (Colzato et al., 2013). Overall, however, the case for proposing that OXT can generally enhance trust in the context of economic games is difficult to support and it should also be noted that they measurement of trust is generally operationalized as willingness to transfer money to another individual who may or may not reciprocate. While studies have indicated that willingness to transfer money in these economic paradigms is significantly associated with levels of interpersonal trust (Van’t Wout and Sanfey, 2008) they are nevertheless independent measures and factors other than altered trust alone could be contributing to OXT influencing investment decision making.

## Oxytocin and Interpersonal Trust in Other Contexts

The effects of intranasal OXT on increasing interpersonal trust have also been investigated in contexts where trust is not measured simply in terms of whether subjects are prepared to give specific individuals more or less money while playing economic games. Mikolajczak et al. (2010b) initially reported that intranasal OXT enhanced trust using an “Envelope Task” paradigm where subjects indicated their level of trust in an experimenter’s promise that their recorded intimate personal details would be kept confidential by whether they wanted to seal the envelope containing their revelations or leave it open. However, this experiment was performed single rather than double blind and failed to be replicated under double-blind conditions (Lane et al., 2015). A few other unpublished studies found no OXT effects on self-reported measures of trust (see Nave et al., 2015) or ones that are dependent upon person-specific characteristics. Thus, one study showed that OXT enhanced levels of trust following social exclusion in the Cyberball game, but only in individuals who reported a negative emotional response to being excluded (Cardoso et al., 2013), and another showed that interpersonal trust was only increased in Democrats with low initial personal trust (Merolla et al., 2013). Finally, one study has reported that intranasal OXT enhanced trust/compliance with reliable, but not unreliable, human-like automatons (De Visser et al., 2017).

The effects of OXT on perception of implicit trustworthiness have also been investigated in a number of other contexts, although again with variable findings. An early study for example reported that intranasal OXT increased trustworthiness ratings of neutral expression faces although this was in combination with attractiveness (Theodoridou et al., 2009) but many studies have subsequently failed to find any effects on facial trustworthiness ratings *per se* (Guastella et al., 2008; Lambert et al., 2014; Quintana et al., 2015; Luo et al., 2017; Woolley et al., 2017), including in either young or old subjects (Grainger et al., 2018). This apparent lack of influence of OXT on ratings of implicit trustworthiness from faces is important given that there is a strong association between such ratings and subjects’ willingness to transfer money to specific individuals in economic games (Van’t Wout and Sanfey, 2008). This would therefore imply that OXT could somehow influence trustworthiness in terms of willingness to invest in another individual although without necessarily making them more implicitly trustworthy. On the other hand, OXT may improve judgments of trustworthiness from faces by enhancing detection of untrustworthy individuals (see Lambert et al., 2014).

Brain imaging studies have shown that perception of trust in faces is negatively associated with activation in limbic regions engaged in fear processing, particularly the amygdala (Engell et al., 2007) and positively associated with activation in core nodes of the reward processing circuitry such as the orbitofrontal cortex and striatum (Mende-Siedlecki et al., 2012). Brain lesion studies have provided additional support for a critical engagement of the amygdala (Adolphs et al., 1998; Koscik and Tranel, 2011; Van Honk et al., 2013), insula (Belfi et al., 2015), and medial prefrontal cortex (Moretto et al., 2013) in trust behavior. The activity and functional connectivity of all of these brain regions have repeatedly been shown to mediate behavioral effects of OXT administration (see Kendrick et al., 2017), although this does not necessarily directly imply that OXT is acting on these same regions to promote trust since they are also implicated more widely in many different aspects of social cognition.

## Oxytocin and Interpersonal Trust in Clinical Populations

In a clinical context trust is of crucial importance in therapist-patient relationships, particularly in therapeutic counseling and behavioral therapy. Our ability to trust others can be impaired as a result of negative social experiences and insecure attachment (Corriveau et al., 2009) and in a number of mental disorders such as schizophrenia (Keri et al., 2009), and some personality disorders such as borderline personality disorder (Fonagy et al., 2015). On the other hand, individuals with disorders, such as Autism Spectrum Disorder may have problems in accurately perceiving trust cues (Yi et al., 2013; Ewing et al., 2015) and therefore find it harder to detect when they are being deceived. However, in the context of clinical studies, intranasal OXT has been found to actually reduce trust in subjects with borderline personality disorder (Bartz et al., 2011a), particularly in individuals with experience of childhood trauma (Ebert et al., 2013). On the other hand, OXT may enhance trust in Prader-Willi syndrome (Tauber et al., 2011), a genetic disorder characterized by disruptive behaviors and marked interpersonal problems. A study on individuals with Autism Spectrum Disorder using a modified Cyberball game paradigm also found that OXT increased co-operation more with individuals who reciprocated throwing the ball back to the subject and they also reported having increased trust in them (Andari et al., 2010). However, use of OXT as an adjunct to behavioral therapy has so far met with limited success, which again could be considered as indirect evidence for it failing to increase trust in the therapist (Guastella et al., 2009; MacDonald et al., 2013).

## Oxytocin and Trust: Summary

Overall therefore, despite the attractiveness and hypothetical support for OXT playing a key functional role in directly influencing interpersonal trust, accumulating empirical evidence makes this view hard to maintain. Indeed, it would seem that as with many behavioral effects of this neuropeptide, there may at the very least be complex contributions of both context and previous personal experience to what precise treatment outcomes on trust perception or behavior are observed (Bartz et al., 2011b; Shamay-Tsoory and Abu-Akel, 2016). In addition, if OXT really does play a fundamental role in promoting interpersonal trust then there would be an expectation that it would not promote behaviors which may serve to damage or weaken trust in some way. In this context recent findings showing that OXT can facilitate envy (Shamay-Tsoory et al., 2009), lying and deception (Shalvi and De Dreu, 2014; Aydogan et al., 2017), including for self-benefit (Scheele et al., 2014; Sindermann et al., 2018) and aggression (Ne’eman et al., 2016) suggest that it can indeed potentially promote trust-damaging behaviors.

## Oxytocin and Social Conformity to Members of In-Groups

We are not only more likely to accept the opinions and advice of others, co-operate with them more and learn from them simply as a result of trusting them more, this can also occur as a result of forming closer social ties with them (Feng and MacGeorge, 2006). It is well established that we will often change our views and preferences to match those expressed by others in our social group in order to fit in. This is referred to as the “social conformity” effect and while it often represents a transient (<3 days) change in our publically expressed views in response either to explicit or implicit social influence (see Huang et al., 2014), it can also result in more enduring changes in our privately held ones. It has been argued that such social conformity reflects learning which is reinforced by the positive reward value of adhering to social norms, together with fear of punishment when we fail to do so (Cialdini and Goldstein, 2004). We are also more likely to accept the advice and opinions of inherently trusted individuals who are part of our social in-group, most notably partners, friends and relatives (Brewer, 2008). We generally trust members of our social in-group more than others and this forms the basis of our increased willingness to co-operate with and protect and learn from them.

Given the evolutionary key role of OXT in promoting affiliative bonds (Kendrick, 2000; Striepens et al., 2011) it may be this aspect of its functioning which increases conformity and willingness to accept and co-operate with and learn from in-group members rather than by enhancing interpersonal trust *per se*. That OXT could function to influence our trust-associated behaviors indirectly by affecting the strength of our affiliation with others at either a group or individual level is firstly supported by another landmark paper in the field. This paper reported that OXT can increase both “trust-in” and “love-for” in-group but not out-group members in the context of monetary investment behavior exhibited during an economic trust game (De Dreu et al., 2010). Further studies have also established that OXT enhances liking for and co-operation with in-group members, irrespective of whether they co-operate with us or not (De Dreu et al., 2011; Ma et al., 2014) and can even increase deceptive behavior for the benefit of in-group members (Shalvi and De Dreu, 2014). A meta-analysis also suggested intranasal OXT elevates the level of in-group but not out-group trust (Van IJzendoorn and Bakermans-Kranenburg, 2012).

Our level of trust in members of our in-group such as friends, family and partners is naturally higher than for members of out-groups and strangers, as is our liking for them, and likability and trustworthiness are correlated to some extent. However, liking and trust are dissociable, with trust for example being associated with levels of perceived self-control in others whereas liking is not (Righetti and Finkenauer, 2011). Liking, attraction and trustworthy judgments from face features are also dissociable and the correlation between liking and trust can be weakened by age (Todorov et al., 2015). Importantly, in the context of establishing the nature of OXT’s relative functional effects on these two social dimensions, studies have more consistently shown that it can enhance liking for the faces of individuals either presented alone (Striepens et al., 2014), or in combination with specific information about an individual’s behavior or expertise (Chen et al., 2015; Gao et al., 2016; Zhao et al., 2018b) rather than trust. Unfortunately, to date only one study has measured both liking and trust following OXT administration and found that it specifically enhanced liking/attraction ratings for individuals and not those of trustworthiness (Xu L. et al., 2018). Here, male and female subjects were required to rate likeability and trustworthiness of potential romantic partners associated with a previous history of fidelity or infidelity and OXT only influenced likeability/attraction ratings and not those of trustworthiness.

In further support of the above proposal, and in marked contrast to the inconsistent findings from studies investigating the effects of OXT on interpersonal trust *per se*, there is substantial and consistent evidence demonstrating that it facilitates conformity to the opinions expressed by groups of in-group members (De Dreu and Kret, 2016). This effect occurs both within culturally long-term established in-groups (Huang et al., 2015) and those formed arbitrarily in the short-term context of a competitive environment (Stallen et al., 2012; Edelson et al., 2015). Furthermore, OXT can also facilitate norm-based compliance and can even counteract ethnocentrism which it normally promotes. This was demonstrated elegantly in the context of individuals who exhibited xenophobic tendencies in terms of charitable donations becoming more likely to donate to immigrants when OXT was administered in association with reinforcement of norm-based altruism (Marsh et al., 2017).

## Oxytocin and Conformity to Perceived “Experts”

Our acceptance that someone is an “expert” of some kind (e.g., elders, teachers, doctors, or professionals in other areas, etc.) implies that we are more likely to consider their opinions and advice in relation to the specific field of their expertise as trustworthy (Bonaccio and Dalal, 2006). As such, we will also learn from and potentially co-operate with them, although this does not necessarily imply that we will consider such individuals as generally more trustworthy than others outside of their area of expertise. In contrast to the situation with in-groups where the effect of OXT on increased conformity and co-operation may be partly contributed to by increased affiliation, that involving similar behavior in relation to perceived experts may be different.

**Figure 1:**
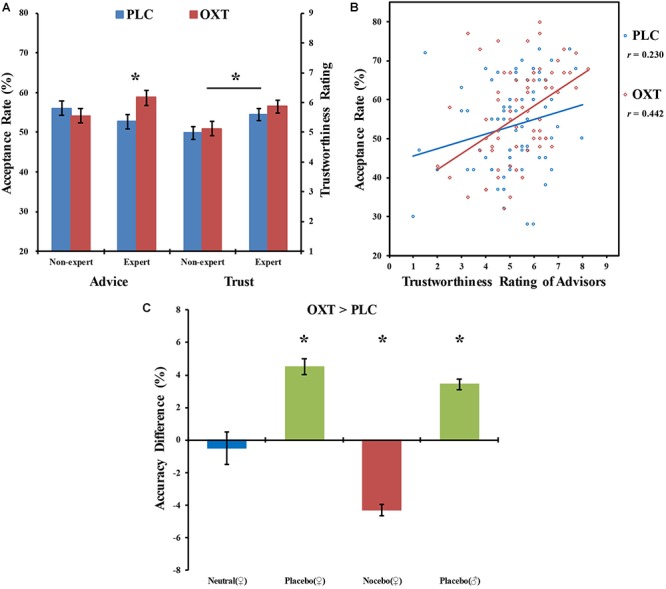
**(A)** Mean ± SEM % acceptance of advice by male subjects on solutions to social problems given by the same female advisors who were either designated as non-experts (landscape gardeners) or experts (psychological counselors) in giving social advice. Before the paradigm participants were randomly assigned to either intranasal oxytocin (OXT – 40 IU) or placebo (PLC) treatment. OXT significantly increased acceptance of advice from the advisor when she was designated as an expert but not a non-expert. While the advisor as an expert was trusted significantly more than as a non-expert, OXT administration *per se* did not influence ratings of trustworthiness. ^∗^*p* < 0.05 for OXT vs. PLC or trust ratings in expert vs. non-expert advisors, respectively. **(B)** Regression graph showing a positive correlation between acceptance of advice from different male and female expert and no-expert advisors and their trustworthiness ratings in subjects receiving PLC or OXT treatment. Subjects receiving OXT generally showed a stronger positive correlation between advice acceptance and trustworthiness ratings [OXT: *r* = 0.442, *p* < 0.001; PLC: *r* = 0.230, *p* = 0.047; Fisher *z*-score = 1.43, *p* = 0.076 (one tailed)]. Data for **(A,B)** are taken from Luo et al. (2017). **(C)** Histograms show the effects of intranasal OXT (24 IU) vs. PLC nasal spray alone (blue bar) or in combination with either advice from a female or male expert in a white coat telling subjects that their working memory performance will be improved (placebo effect, green bar) or impaired (nocebo effect, red bar). Results are combined data from verbal, spatial, and social n-back tasks (1-back and 2-back combined) taken from Zhao et al. (2018a). ^∗^*p* < 0.05 OXT vs. PLC.

Recently we have demonstrated that OXT-enhancement of conformity can extend to the context of acceptance of social advice from individual experts in psychological counseling (Luo et al., 2017). In this study, male participants were first invited to provide solutions to a number of everyday social problems and then following treatment were given alternative advice (either better or worse) by male or female experts or non-experts (landscape gardeners). Participants were not familiar with the advisors and were simply shown pictures of them together with information about their expertise. All the advisors were older than the subjects to further enhance their perceived experience and potential reliability (see Lourenco et al., 2015). Oxytocin treatment significantly increased the proportion of advice accepted from female experts, irrespective of whether the solutions offered by them were better or worse than those originally chosen by the subjects themselves (see Figure 1A). The use of a counterbalanced design ensured that this effect of OXT was independent of the appearance of specific advisors and therefore influenced only by their attributed expertise. Importantly, OXT did not influence the perceived trustworthiness or likeability of either the experts or the non-experts, but its effects on acceptance of advice were positively associated with both (see Figure 1B). This resulted in a greater degree of acceptance of advice from female experts who were generally rated as more trustworthy and likeable than both female non-experts and the equivalent male experts. Indeed, overall acceptance of advice across all advisors was positively correlated with their perceived trustworthiness and OXT tended to increase this correlation (see Figure 1B). Thus, the study provides the first direct evidence for an interaction between perceived trustworthiness and likeability and the ability of OXT to increase conformity to advice given by individual experts. While both this study and one from another group (Edelson et al., 2015) showed that the effect of OXT on increasing conformity was transient, this is perhaps not that surprising given the rather controlled contexts and that advice is given only once. Indeed, we don’t tend to give up self-held beliefs and judgments very easily and sometimes will deliberately disobey expert advice (Engelmann et al., 2009; Suen et al., 2014). Further experiments are required to investigate whether OXT can influence long-term privately held views following repetition of advice in more natural circumstances and the extent to which it can alter the behavior of individuals who tend to disobey expert advice.

While studies have yet to establish the neural substrates where OXT may act to facilitate taking advice from experts the amygdala may be of importance in this respect. The amygdala shows increased activation during the positive evaluation of advisor competence (Schilbach et al., 2013) and following advice (Biele et al., 2011) and the amygdala is also one of the primary regions where OXT has been found to produce functional effects on social cognition (Kendrick et al., 2017).

That OXT may act to enhance conformity to, and co-operation with, individuals who are highly trusted is consistent with the repeated observations that it facilitates these behaviors in members of an in-group, who are perceived as more trustworthy and likeable than out-group members. Within the context of in-groups, however, OXT may also enhance the perceived expertise of some individuals compared to others without necessarily influencing their trustworthiness. Thus in pairs of subjects working together to solve a visual search task intranasal OXT treatment made the less competent partner more likely to conform to the opinion of the more competent one, and had the opposite effect on the competent partner (Hertz et al., 2016). The perceived expertise factor can also help explain why OXT increases conformity with out-group members in some contexts. For instance, when male Chinese subjects were asked to judge the attractiveness of Asian female faces, and then informed about ratings given by male peers from an in-group (Chinese) or an out-group (Japanese), OXT increased conformity to the opinions of both (Huang et al., 2015). In contrast, OXT increased the likeability of Chinese people, monuments and commercial products but did not have any effect on liking/disliking of comparable Japanese stimuli, suggesting that it reliably induces an in-group preference within this context (Ma et al., 2014). Arguably, male peers from both Chinese and Japanese cultures would be considered to have similar expertise in judging the facial attractiveness of Asian female faces and this perceived similarity of expertise may have resulted in OXT enhancing the impact of the opinions of both due to equivalent levels of trust in the expertise of in-group and out-group members in this specific context.

Other contexts in which OXT appears to function to enhance acceptance of information or skills provided by trusted experts is in relation to its facilitation of placebo effects and also susceptibility to hypnosis. Several studies have now demonstrated that intranasal OXT can enhance or even generate placebo effects. Thus, in the context of analgesia OXT has been reported to enhance the placebo effect on pain perception (Kessner et al., 2013). In a recent experiment we also showed that OXT given in conjunction with a male or female experimenter wearing a white coat informing them that the treatment would enhance their working memory performance exhibited an impressive 5% increased improvement in accuracy in verbal, spatial, and social domains (Zhao et al., 2018a). In the absence of the adjunct OXT treatment there was no placebo effect and no effect of OXT given alone. Importantly, OXT could also generate an equivalent magnitude nocebo effect where subjects informed that the treatment would make them perform worse rather than better did indeed show an equivalent 5% significant performance deficit (Zhao et al., 2018a; see Figure 1C). Furthermore, intranasal OXT can increase the hypnotizability of individuals normally showing low pre-treatment susceptibility to hypnosis, although without influencing their perceived trust in the hypnotist (Bryant et al., 2012). Together these findings further demonstrate that OXT can facilitate the impact of advice/information received from individual experts without necessarily making such individuals either more likeable or trustworthy. Indeed, we have recently reported that OXT can promote increased co-operation with individuals in the Cyberball game who they rate as both less trustworthy and likeable due to their exclusion of other players in order to gain a higher monetary reward (Xu X. et al., 2018). In this case subjects receiving OXT played more with these specific players since doing so would be likely to increase their own financial gain, i.e., there would be a greater expectation that such excluder players would reciprocate with them for mutual benefit despite them being considered generally less trustworthy and likeable.

## Oxytocin as a Facilitator of Social Learning

Overall therefore, it may be more relevant to consider OXT as functioning to facilitate social learning both as a result of enhancing affiliative bonds with trusted in-group members and also from the information/advice/skills transmitted by trusted experts who are not necessarily within an individual’s immediate social group. Trust in these two contexts may potentially be more “affective” in the in-group context and more “cognitive” in the expert one. While distinctions between the relative importance of cognitive and affective trust are made routinely in economic and business contexts (see Dowell et al., 2015) they are not usually distinguished in interpersonal social ones, and this may be important when considering effects of OXT in light of it facilitating emotional empathy but not cognitive empathy in some tasks (Hurlemann et al., 2010; Geng et al., 2018).

Social learning from trusted individuals and groups plays a fundamental role throughout our lives in both promoting social cohesion as well as providing us with the information and strategies to cope with and adapt to the challenges we face every day. While social learning has often been considered as distinct from other forms of learning it has been shown to involve the same associative processes as simple reward-based learning (Behrens et al., 2008). There is increasing evidence that OXT may be playing a key role in promoting social learning from the most appropriate individuals and several studies have also demonstrated this in the context of enhanced probabilistic learning of arbitrary information following social but not non-social reinforcement (Hurlemann et al., 2010; Hu et al., 2015). In these two latter studies subjects were required to learn which of a group of random 3-digit numbers was arbitrarily associated with two different categories following receipt of either social (smiling vs. angry faces) or non-social (red vs. green traffic lights) feedback. OXT selectively enhanced learning with social, but not non-social, feedback in both Caucasian (Hurlemann et al., 2010) and Asian (Hu et al., 2015) subjects and this was associated with increased activation in the amygdala and striatal regions and their functional connectivity (Hu et al., 2015). These studies were unable to distinguish whether OXT differentially enhanced the effects of positive and/or negative social feedback, however, another group demonstrated that OXT enhances activity in the ventral tegmental area in response to both positive (smiling faces) and negative (angry faces) feedback (Groppe et al., 2013). Thus, and in line with social conformity being reinforced by both social reward and fear of social punishment, it seems likely that OXT is promoting social learning via not only increasing the impact of positive social reward cues but also those of social punishment via modulation of amygdalo-frontal-striatal reward networks.

In general reward-based learning involves two different decision control systems, a cognitive “model-based” system and a simpler “model-free” system based on habit (Dolan and Dayan, 2013). Both model-based and model free learning engage striatal circuitry (Daw et al., 2011) and therefore OXT could potentially influence both, although to date only simple probabilistic model-free learning paradigms have been used which guide action and do not involve any rule learning but can easily be utilized to compare the relative effects of social compared with non-social feedback.

## Clinical Implications of Oxytocin as a Facilitator of Social Learning

In support of an important role for OXT in promoting social learning in a clinical context a recent study has reported that it facilitates probabilistic learning with social feedback and enhanced striatal activation in individuals with Autism Spectrum Disorder (Kruppa et al., 2018). This study used the same learning paradigm where OXT was found to facilitate learning with social feedback in healthy subjects although only positive and neutral social feedback were included. In terms of the potential therapeutic use of intranasal OXT to improve the efficacy of cognitive or other therapist-based interventions for mental disorders there are several implications of the findings and interpretations detailed in the current review. Firstly, there is no convincing evidence that OXT treatment *per se* will make individuals more trustworthy, although it may do so indirectly by strengthening affiliative ties with in-group members. While trust in a specific therapist might effectively increase over time as they become equivalent to an in-group member for individual patients, clearly under the majority of circumstances it would be greater importance if patients have a high level of trust in the ability of the therapist as an expert if adjunct OXT treatment is likely to have any beneficial effect. If patients are suspicious of, or have low levels of trust in, a therapist then either OXT is unlikely to have any beneficial effect or quite possibly it might end up having a negative impact by further reducing trust levels, as for example observed in borderline personality disorder (Bartz et al., 2011a; Ebert et al., 2013). Thus, an important consideration for whether OXT might be beneficial as an adjunct to any kind of therapist-based behavioral intervention may be a patient’s general levels of interpersonal trust and trust in their specific therapist. This underlines the well-established importance of initial trust-building between patient and therapist termed the “working-alliance,” the strength of which has a strong bearing on treatment outcome and patient satisfaction (Fuertes et al., 2007).

## Conclusion and Future Directions

In summary, we have argued in this review that OXT administration rather than enhancing either implicit or explicit trust in others is instead primarily promoting social cohesion by facilitating increased conformity to others who we trust either as in-group members and/or as perceived experts. As such, OXT can be viewed as facilitating socially reinforced learning, particularly from trusted individuals, via amygdalo-frontal-striatal circuitry to increase the motivation to receive and respond to either social reward or punishment. It will be important in future studies to establish how cognitive and emotional aspects of trust interact with the effects of OXT on social learning and conformity. To date the effects of OXT have also only been investigated in the context of simple reinforcement paradigms involving model-free learning and it will be important to investigate whether they can extend to more cognitive model-based learning. From a therapeutic standpoint it will also be important in future studies to determine the extent to which levels of patient trust in the expertise of the therapist influence the effectiveness of OXT as an adjunct to behavior therapy.

## Author Contributions

All authors contributed to the information and ideas presented in the review and writing of the manuscript.

## Conflict of Interest Statement

The authors declare that the research was conducted in the absence of any commercial or financial relationships that could be construed as a potential conflict of interest.
